# Supplementary material for: Generation of a single-cell B cell atlas of antibody repertoires and transcriptomes to identify signatures associated with antigen specificity
Source: iScience. 2023 Jan 25;26(3):106055. doi: 10.1016/j.isci.2023.106055 (PMC9958373; doi:10.1016/j.isci.2023.106055)
Supplement: Document S1. Figures S1–S13 [file mmc1.pdf]

iScience, Volume *26*

Supplemental information

Generation of a single-cell B cell atlas of antibody repertoires and transcriptomes to identify signatures associated with antigen specificity

Andreas Agrafiotis, Daniel Neumeier, Kai-Lin Hong, Tasnia Chowdhury, Roy Ehling, Raphael Kuhn, Ioana Sandu, Victor Kreiner, Tudor-Stefan Cotet, Danielle Shlesinger, Daria Laslo, Stine Anzböck, Dale Starkie, Daniel J. Lightwood, Annette Oxenius, Sai T. Reddy, and Alexander Yermanos

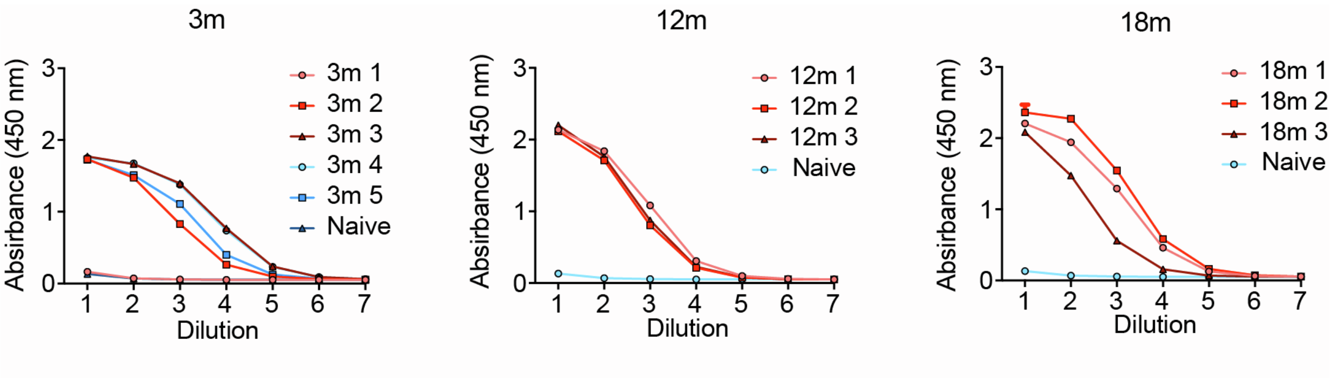

Figure S1. Serum antibody titers against hTNFR2 for each age group, Related to Figure 1. Each line corresponds to a mouse. A naive mouse was included as a control.

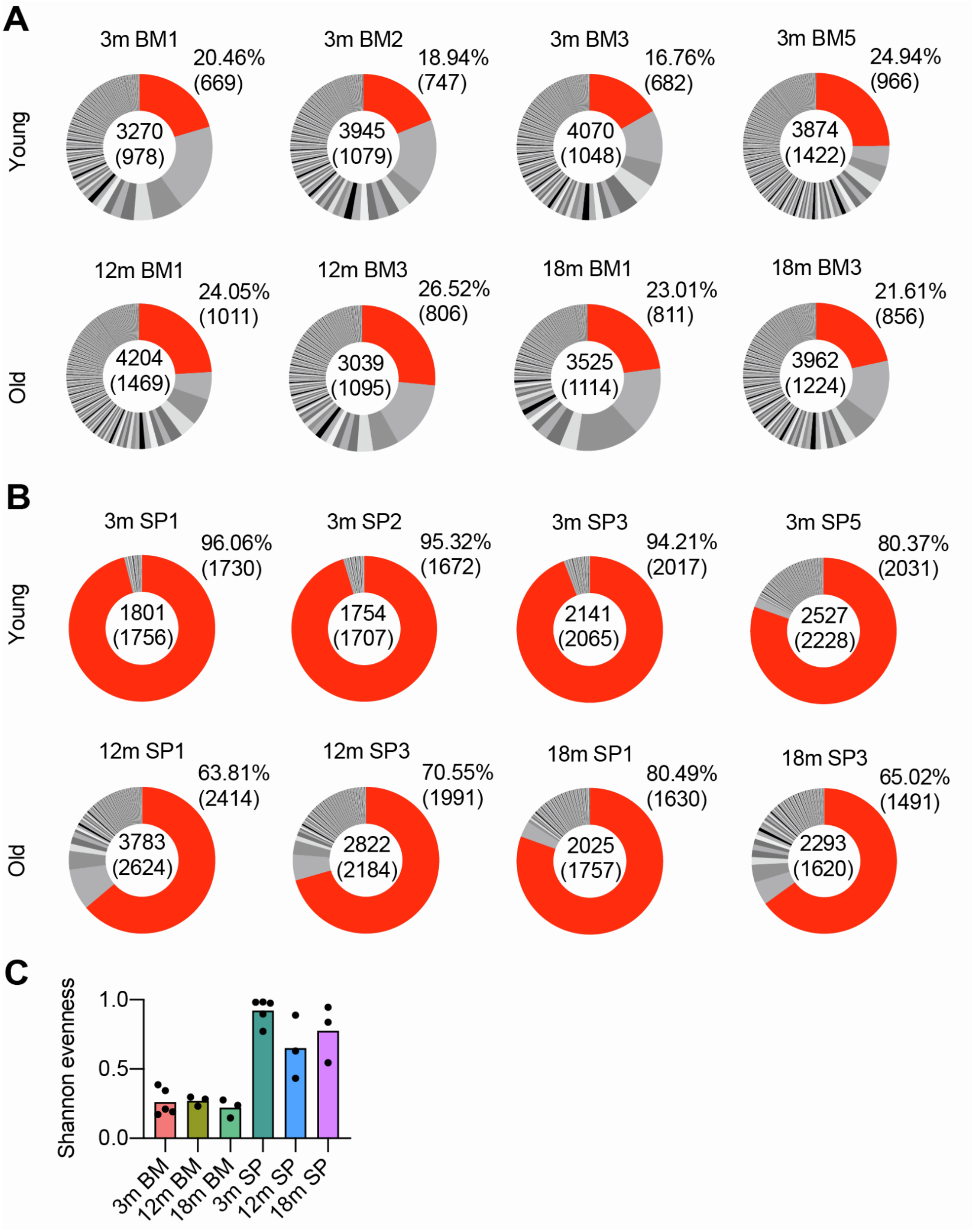

Figure S2. Clonal expansion following TNFR2 immunization, Related to Figure 1. Distribution of clonal expansion in the A. bone marrow (BM) and B. spleen (SP). Each section corresponds to a unique clone and the size corresponds to the fraction of cells relative to the total repertoire. Red color highlights the fraction of clones containing 1 cell. Numbers in the center indicate the total number of cells and clones (in parenthesis). Numbers on the right indicate the percentage and total number (in parenthesis) of unexpanded clones. C. Shannon evenness quantifying clonal expansion of BM PCs and splenic B cells. Each individual point corresponds to a repertoire arising from a 3-month-old (3m), 12-months-old (12m), or 18-month-old (18m) mouse. Colors correspond to the different age and organ cohorts.

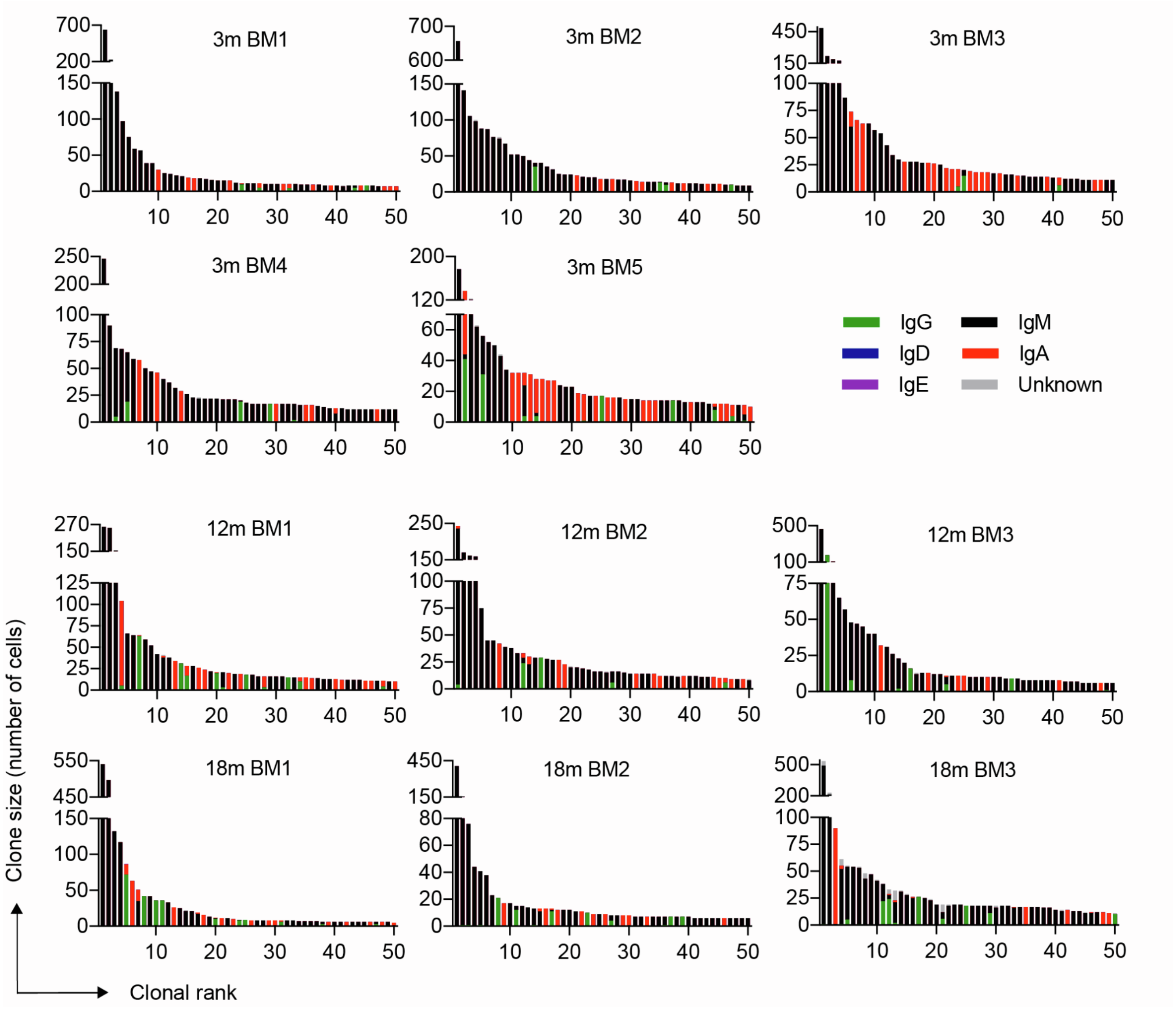

Figure S3. Clonal expansion for the top 50 most expanded clones of the BM PCs for each immunized mouse, Related to Figure 1. Clones were determined by grouping those B cells containing identical CDRH3+CDRL3 amino acid sequences. Color corresponds to isotype. Each plot corresponds to a repertoire arising from a 3-month-old (3m), 12-months-old (12m), or 18-month-old (18m) mouse.

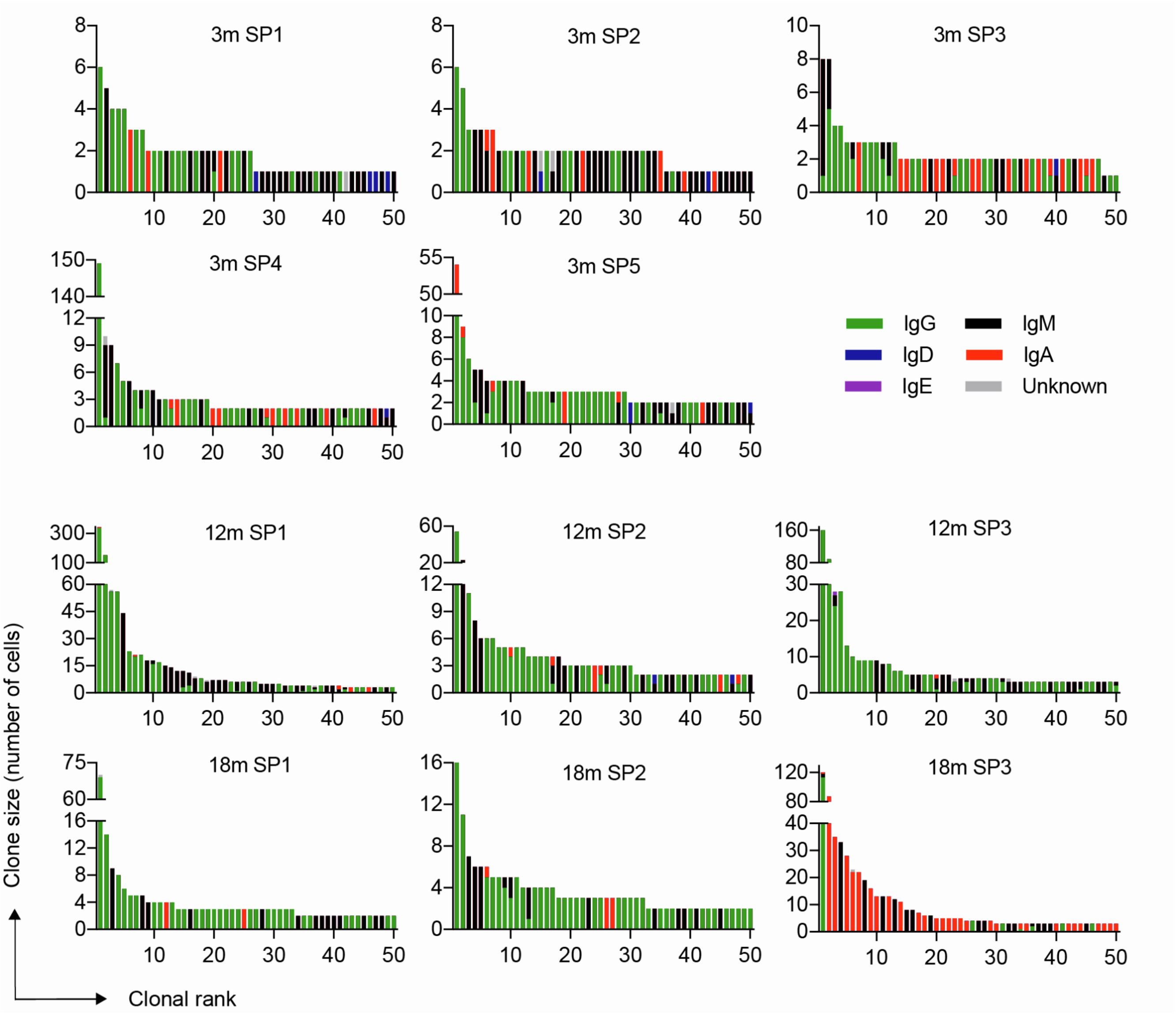

Figure S4. Clonal expansion for the top 50 most expanded clones of the splenic (SP) B cells for each immunized mouse, Related to Figure 1. Clones were determined by grouping those B cells containing identical CDRH3+CDRL3 amino acid sequences. Color corresponds to isotype. Each plot corresponds to a repertoire arising from a 3-month-old (3m), 12-months-old (12m), or 18-month-old (18m) mouse.

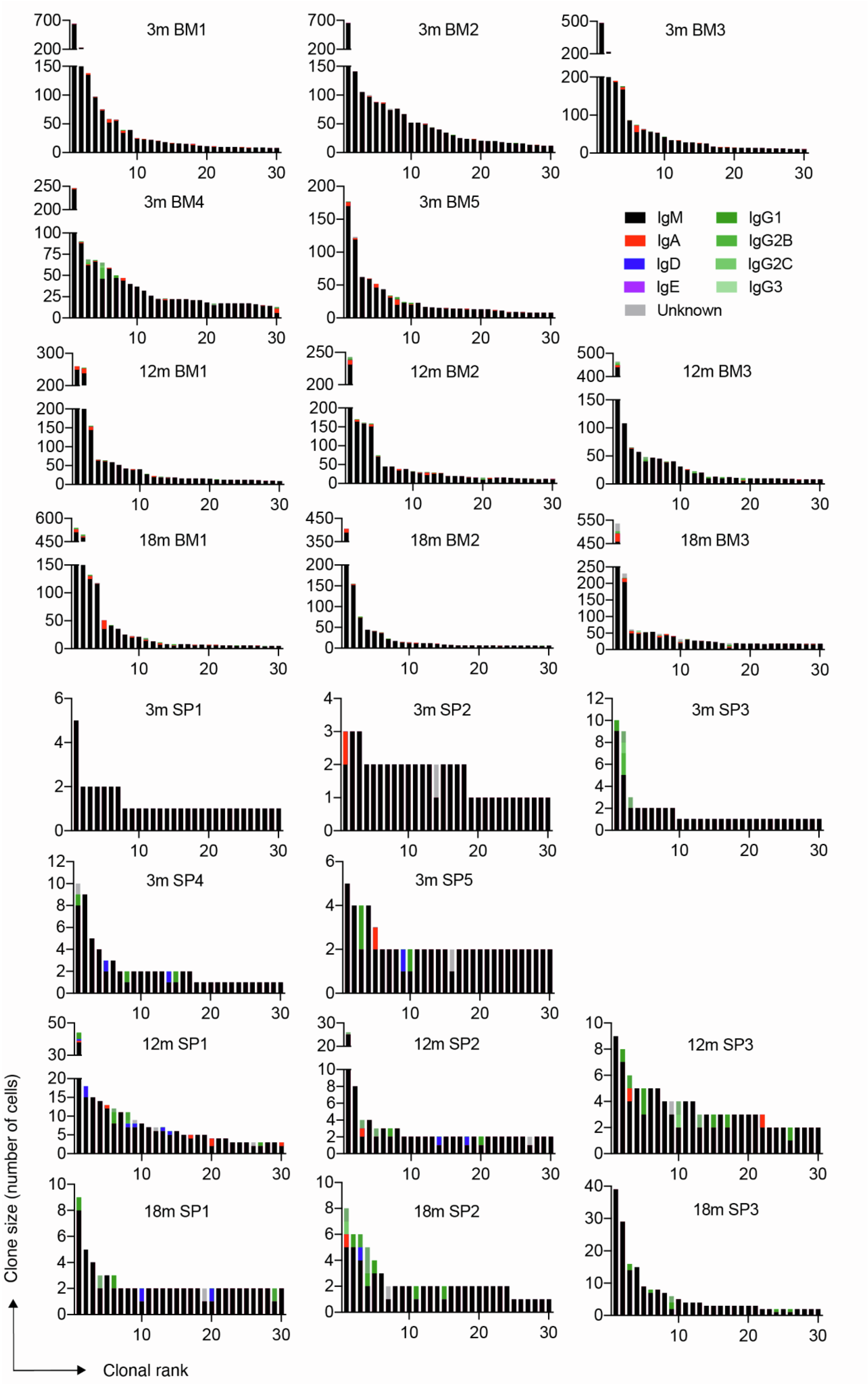
3m BM1
3m BM2
3m BM3
3m BM4
3m BM5
IgM
IgA
IgD
IgE
Unknown
IgG1
IgG2B
IgG2C
IgG3
12m BM1
12m BM2
12m BM3
18m BM1
18m BM2
18m BM3
3m SP1
3m SP2
3m SP3
3m SP4
3m SP5
12m SP1
12m SP2
12m SP3
18m SP1
18m SP2
18m SP3
Clone size (number of cells)
Clonal rank

Figure S5. Clonal expansion for the top 30 most expanded clones with the majority of cells belonging to the IgM isotype of the BM PCs and splenic B cells for each immunized mouse, Related to Figure 1. Clones were determined by grouping those B cells containing identical CDRH3+CDRL3 amino acid sequences.

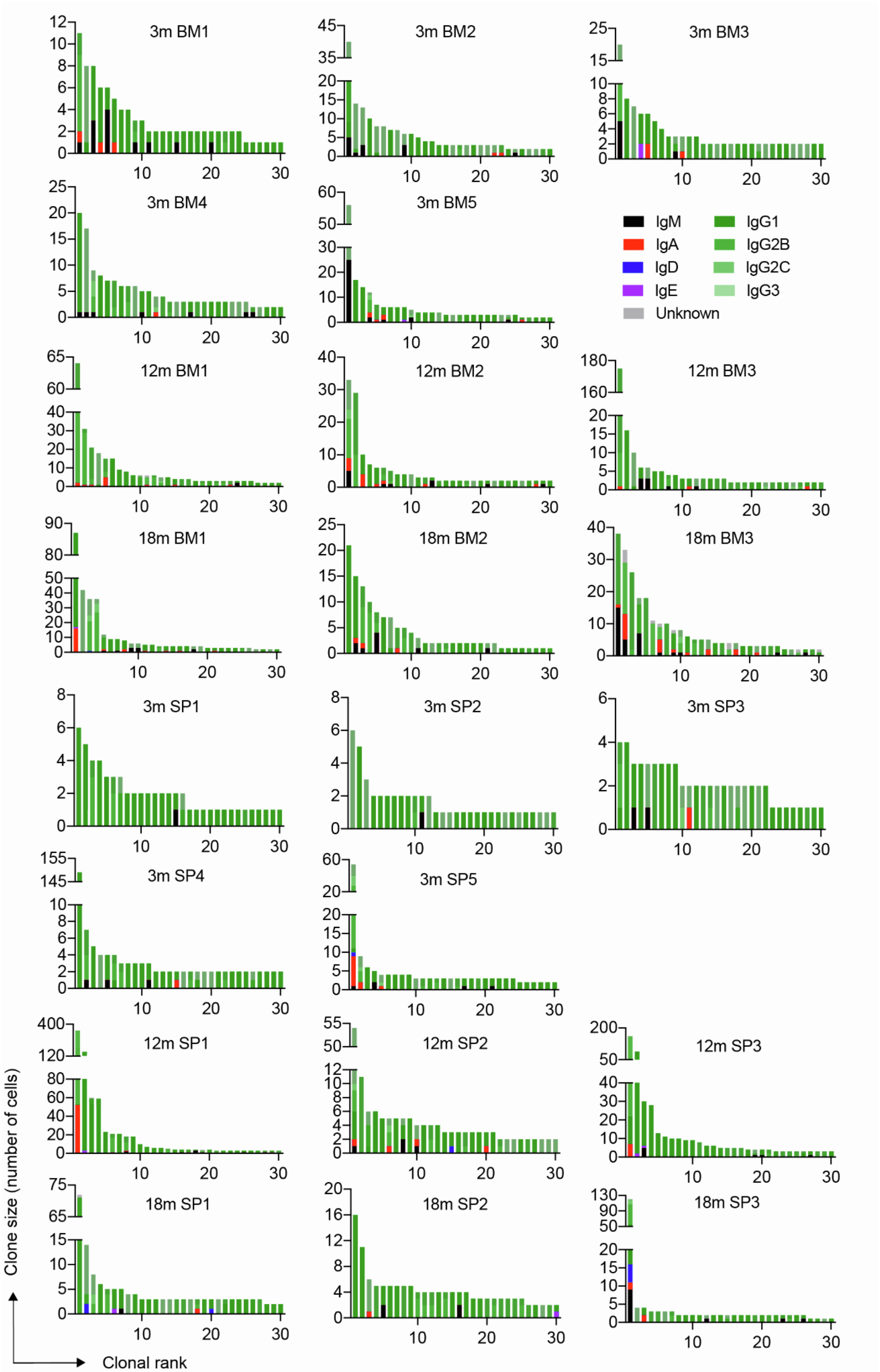

3m BM1
3m BM2
3m BM3
3m BM4
3m BM5
IgM
IgA
IgD
IgE
Unknown
IgG1
IgG2B
IgG2C
IgG3
12m BM1
12m BM2
12m BM3
18m BM1
18m BM2
18m BM3
3m SP1
3m SP2
3m SP3
3m SP4
3m SP5
12m SP1
12m SP2
12m SP3
18m SP1
18m SP2
18m SP3
Clone size (number of cells)
Clonal rank

Figure S6. Clonal expansion for the top 30 most expanded clones with the majority of cells belonging to the IgG isotype of the BM plasma and splenic B cells for each immunized mouse, Related to Figure 1. Clones were determined by grouping those B cells containing identical CDRH3+CDRL3 amino acid sequences.

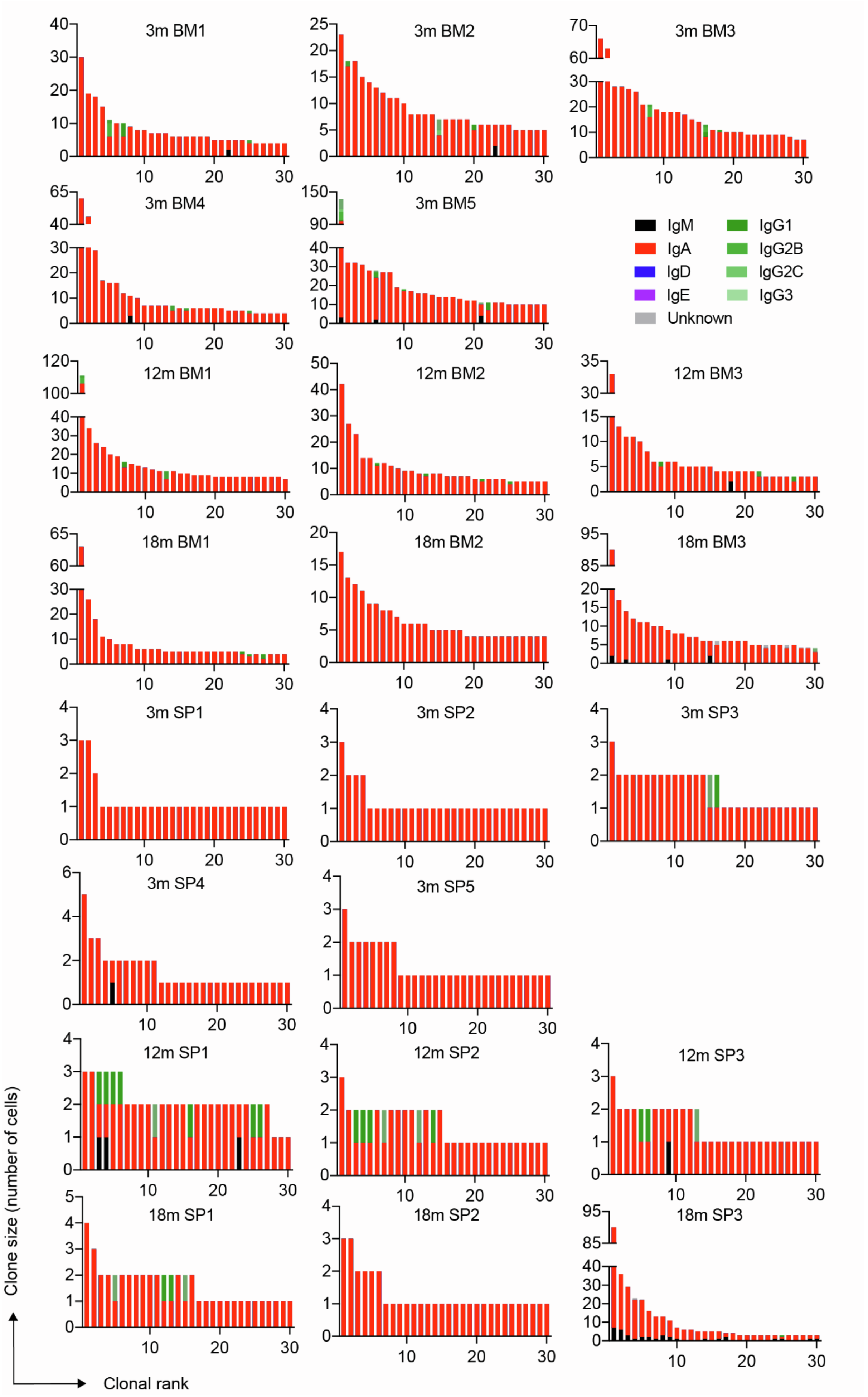
3m BM1
3m BM2
3m BM3
3m BM4
3m BM5
12m BM1
12m BM2
12m BM3
18m BM1
18m BM2
18m BM3
3m SP1
3m SP2
3m SP3
3m SP4
3m SP5
12m SP1
12m SP2
12m SP3
18m SP1
18m SP2
18m SP3
IgM
IgA
IgD
IgE
Unknown
IgG1
IgG2B
IgG2C
IgG3
Clone size (number of cells)
Clonal rank

Figure S7. Clonal expansion for the top 30 most expanded clones with the majority of cells belonging to the IgA isotype of the bone marrow plasma and splenic B cells for each immunized mouse, Related to Figure 1. Clones were determined by grouping those B cells containing identical CDRH3+CDRL3 amino acid sequences.

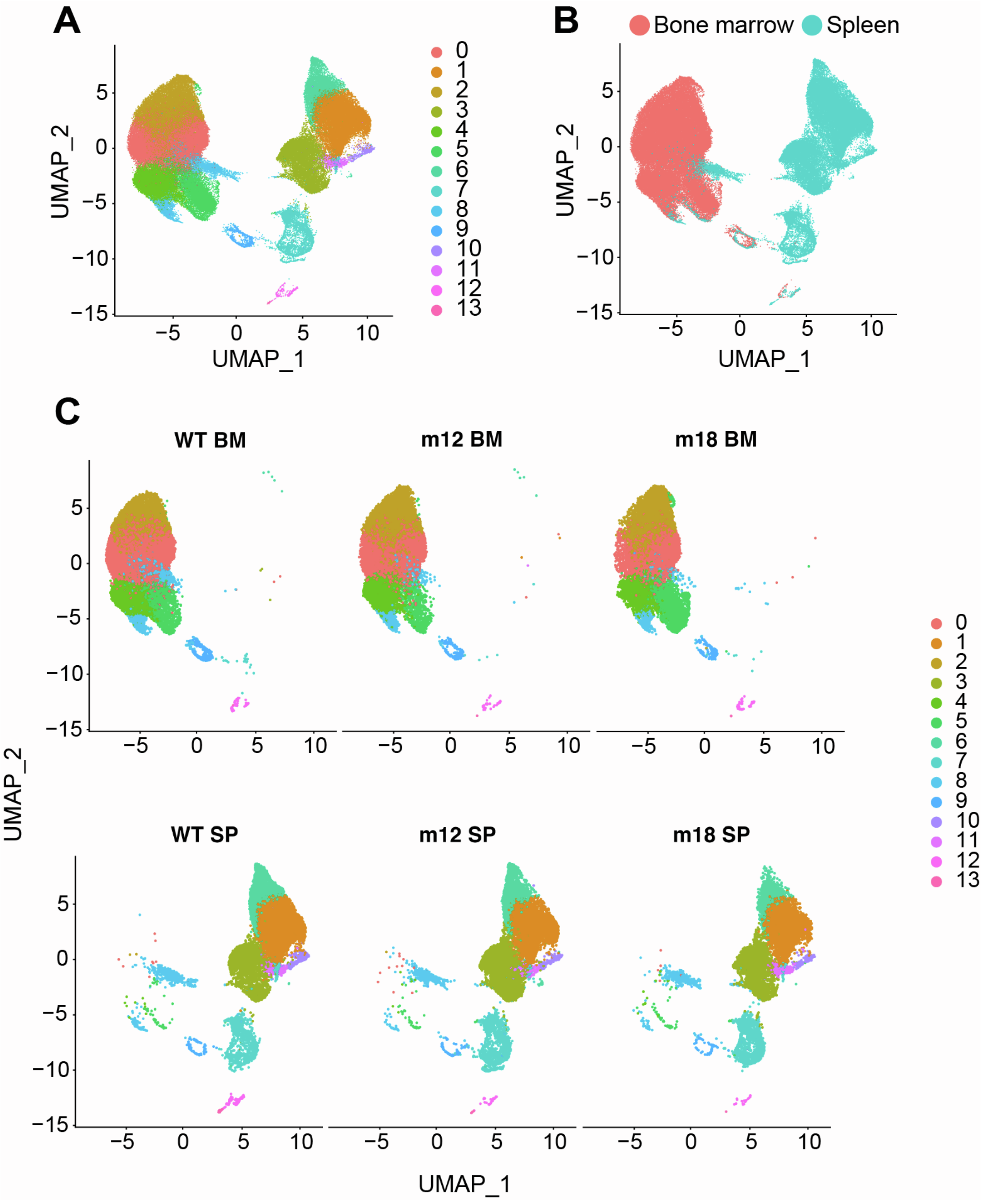

Figure S8. Single-cell transcriptome sequencing of B cells, Related to Figure 2. A. Uniform manifold approximation projection (UMAP) based on total gene expression of all repertoires following TNFR2 immunization. Each point corresponds to a cell and color corresponds to the transcriptional cluster. B. UMAP split by organ. C. Uniform manifold approximation projection (UMAP) split by age and organ cohort. Each point corresponds to a cell and color corresponds to the transcriptional cluster.

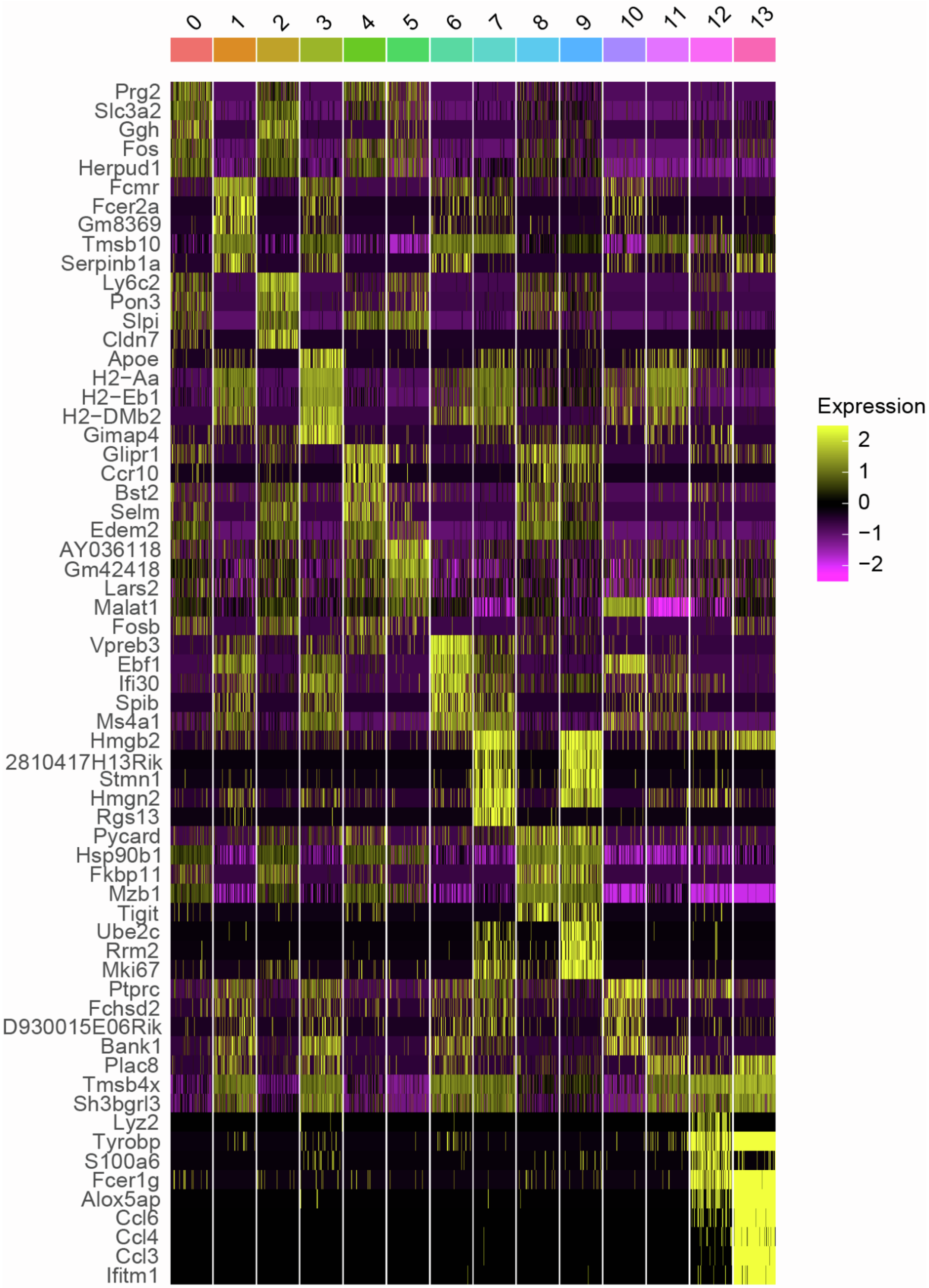

Figure S9. Top differentially expressed genes for each transcriptional cluster for all repertoires, Related to Figure 2. Heatmap intensity corresponds to normalized expression. Each column represents a single cell and each row corresponds to a single gene. The top five genes based on average log fold change (avg_log2FC) have been

selected for each cluster after removing ribosomal protein L (RPL), ribosomal protein S (RPS), and mitochondrial genes. All displayed genes had an adjusted p value less than or equal to 0.01.

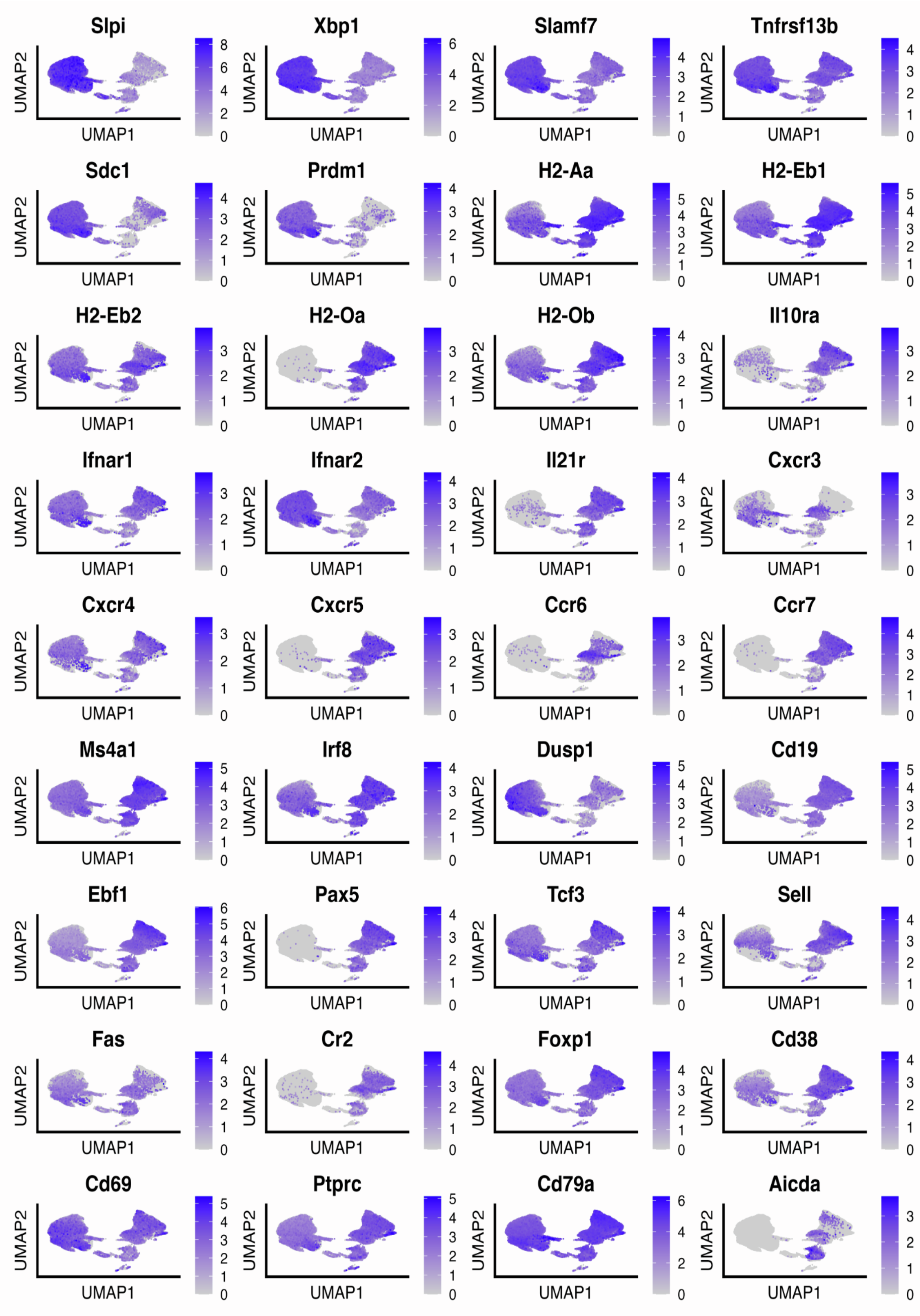
Slpi
Xbp1
Slamf7
Tnfrsf13b
Sdc1
Prdm1
H2-Aa
H2-Eb1
H2-Eb2
H2-Oa
H2-Ob
Il10ra
Ifnar1
Ifnar2
Il21r
Cxcr3
Cxcr4
Cxcr5
Ccr6
Ccr7
Ms4a1
Irf8
Dusp1
Cd19
Ebf1
Pax5
Tcf3
Sell
Fas
Cr2
Foxp1
Cd38
Cd69
Ptprc
Cd79a
Aicda
UMAP1
UMAP2

Figure S10. Uniform manifold approximation projection (UMAP) plots showing gene expression for selected genes, Related to Figure 2.

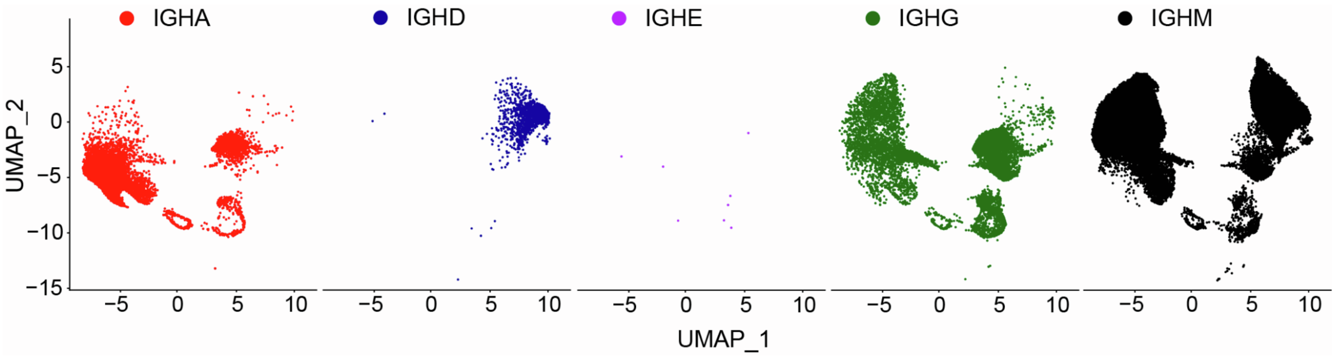

Figure S11. Uniform manifold approximation projection (UMAP) split by isotype, Related to Figure 2. Each point corresponds to a cell and color corresponds to the respective isotype.

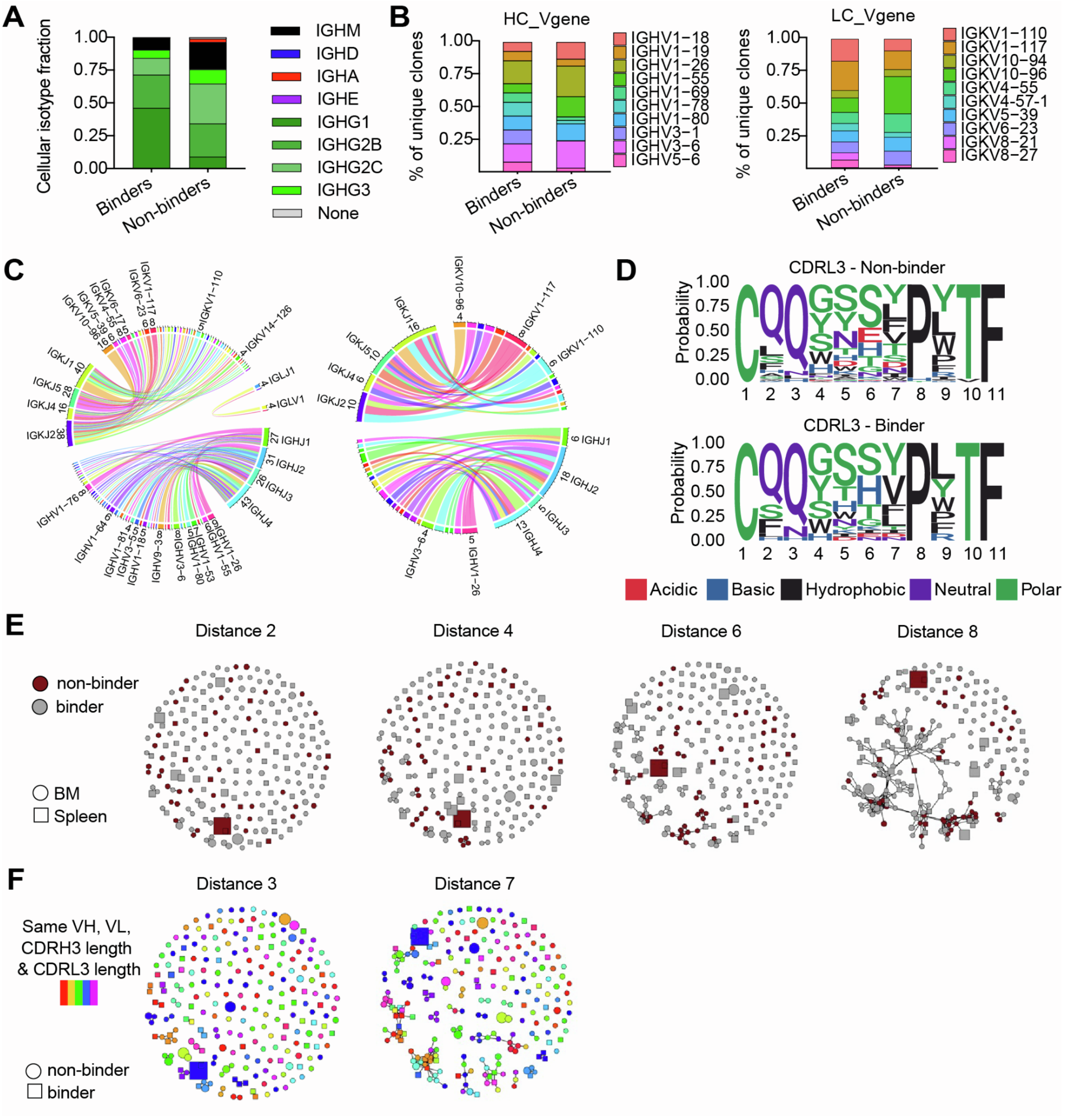

Figure S12. Immune repertoire and transcriptional features of TNFR2-specific sequences, Related to Figure 4. A. Isotype distribution for the confirmed bone marrow TNFR2-specific and non-specific sequences on the cellular level. B. Sequence logo plots of the confirmed TNFR2-specific and non-specific CDRL3 sequences for the most frequent CDRL3 length. C. Heavy chain (right) and light chain (left) V gene distribution between antigen-specific and non-specific clones. D. Circos plots depicting the relationship between HC and LC V and J genes for TNFR2 specific (left) and non-specific (right) sequences in the BM. Color corresponds to the different V genes. Edges illustrate the number of clones using each particular combination. Only the most frequent V genes are noted. E. Similarity network of TNFR2-specific and non-specific clones. Nodes represent a unique clone. Edges connect those clones separated by an edit distance of 2, 4, 6 and 8 amino acids or less. Color corresponds to TNFR2 specificity and shape indicates organ of origin. F. Same similarity network as (E) with color indicating same variable HC, variable LC, CDRH3 length and CDRL3 length. Shape indicates TNFR2 specificity.

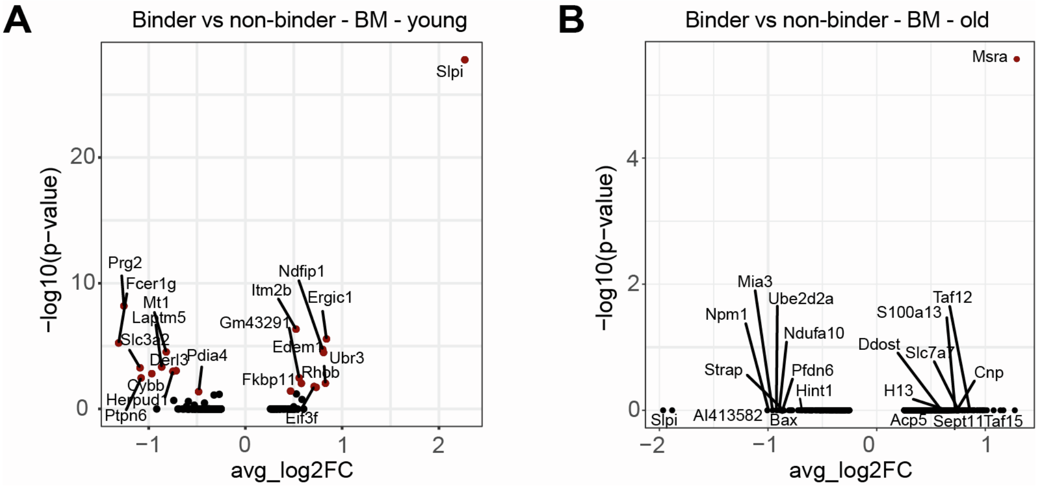

Figure S13. Differential gene expression between TNFR2 specific and non-specific clones in the bone marrow of young (A) and old (B) mice, Related to Figure 4. Points in red indicate significantly differentially expressed genes (p-adj < 0.01).
